# What to eat for a better sleep in haemodialysis patients: Potential role of B vitamins intake and appetite

**DOI:** 10.12669/pjms.332.11838

**Published:** 2017

**Authors:** Dilek Ongan, Aysun Yuksel

**Affiliations:** 1 Dr. Dilek Ongan, PhD, Department of Nutrition and Dietetics, Faculty of Health Sciences, Izmir Katip Celebi University, Izmir, Turkey; 2 Dr. Aysun Yuksel, PhD, Izmir Public Health Directorate, Buca Public Healthcare Center Izmir, Turkey

**Keywords:** Appetite, Haemodialysis patients, Nutritional status, Pittsburgh Sleep Quality Index, Sleep quality

## Abstract

**Objective:**

Sleeping disorders are common among Haemodialysis-HD patients. In addition to well-known factors, food consumption impact on sleep quality needs being discovered. Aim was to examine the nutrition-related factors that may influence sleep quality in HD patients.

**Methods:**

One hundred and three patients in three HD centres participated. Data were collected with Pittsburgh Sleep Quality Index-PSQI and Questionnaire Form about socio-demographic characteristics and appetite. Biochemical findings were obtained; food consumption for three consecutive days was recorded. Independent Samples t/Mann Whitney U tests for mean comparison; Logistic Regression Analysis for determining variables affecting sleep quality, were used.

**Results:**

Mean age was 59.19±14.57 years. 51.5% were women. 37.9% had good (PSQI<5) and 62.1% had poor (PSQI≥5) sleep quality. Appetite had significant impact on sleep quality; poor sleeping risk was 4.194 fold higher in patients with bad appetite than those with good appetite (p=0.038). Biochemical findings of poor and good sleepers were similar, except for potassium and creatinine. Vitamins B_1_, B_6_ and folate intake of good and poor sleepers were significantly different. Patients with good sleep quality consumed significantly higher amounts of above B vitamins (p=0.030, p=0.036, p=0.034, respectively).

**Conclusions:**

Favourable effect of appetite and certain nutrients intake on sleep quality in HD patients was shown. Improving nutritional status of HD patients has potential to increase their sleep quality.

## INTRODUCTION

Chronic Kidney Disease (CKD) has become globally epidemic due to increased life duration and diabetes-hypertension cases.^1^ These patients have sleeping problems which decrease quality of life besides dietary restriction, nutritional problems, HD treatment.^2^ Sleeping disorders among HD patients is prevalent (50-83%). Metabolic alterations, pain, dyspnea, fatigue, cramp, and peripheral neuropathy cause sleeping problems. In patients with CKD, abnormal cellular interleukin production occurs which causes sleepiness however HD causes sleeping problems by excretion of this sleep-inducing substance.^3^ Circadian rhythm deteriorates in HD patients because melatonin secretion, which plays the crucial role in biological clock and circadian sleep-wake rhythms and in the onset of the sleep in evenings, decreases when the kidney functions decline. Moreover, nocturnal melatonin secretion was reported to be absent in HD patients.^4^ Uremic restless leg syndrome (URL-Synd) negatively affects sleep. Parathyroid hormone levels may also be associated with poor sleep.^5^ Tryptophan reducing enzyme indoleamine-2,3-dioxygenase causes tryptophan catabolites increase in HD patients. Inflammatory processes stimulate tryptophan degradation which then triggers sleep disorders.^6^ Insufficient sleep in CKD patients is known to cause three important complications (Type 2 Diabetes Mellitus-T2DM, hypertension, obesity).^7^

Besides above causes, poor appetite which influences patients’ food consumption and nutritional status may have unfavourable effect on sleep. In HEMO cohort, sleep quality of patients was associated with appetite and serum creatinine levels, showing that the more appetite is lost, the more sleep quality score decreases.^8^ Patients with good sleep quality were found to eat more meat, cheese, egg and fruits than those with poor sleep quality while albumin, pre-albumin levels and total lymphocyte count were significantly lower of bad sleepers.^9^ Dietary protein intake, serum albumin concentrations and appetite were determined as crucial predictors of sleep by Burrowes et al.^10^ Monitoring nutritional status and appetite of HD patients are important care standards,^11^ however its association with sleep quality is not yet well documented. Therefore this study was planned to examine the nutrition-related factors that may influence patients’ sleep quality, in addition to finding out possible dietary implementations for HD patients with poor sleep by excluding the other sleep-deteriorating factors.

## METHODS

### Study Population and Sample Size

In this study, 103 voluntary HD patients, who were being treated in HD centres in three State Hospitals of Eskişehir/Turkey, were recruited. According to power analysis from Köse et al’s^9^ study (effect size=7,1, alpha=0,05, statistical power=0,80); sample was calculated as 102 patients.

### Inclusion Criteria

Being on HD treatment for at least three months; for at least three sessions a week; older than 18 years old.

### Exclusion Criteria

Having Chronic Obstructive Pulmonary Disease, sleep apnoea, neoplastic diseases, URL-Synd, psychiatric diseases taken anti-depressives and sedatives.

### Data Collection

Data were collected with questionnaire form about socio-demographic characteristics and PSQI. Appetite was determined with subjective evaluation of its level (“my appetite is very bad/bad/moderate/good/very good”). Biochemical findings (fasting blood glucose, sodium, potassium, calcium, phosphorus, blood urea nitrogen-BUN, serum creatinine, uric acid, iron, haemoglobin, haematocrit, total cholesterol, HDL-cholesterol, LDL-cholesterol, protein, serum albumin) were obtained from routine monthly analysis reports of the same hospital laboratory. Food intake was assessed with dietary records for three consecutive days.

### Assessment of Sleep Quality

Sleep quality was assessed with PSQI. As a consistent, replicable and reliable index, PSQI is used to determine sleep quality during last month.^12^ PSQI provides evaluating quality and amount of sleep, existence of sleep disorder and its severity. PSQI, which has 19 self-rating questions and 5 spouse/roommate-answered questions, was applied with face-to-face interview. Seven items of PSQI evaluate subjective quality, delay, efficiency, duration of sleep, use of sleeping pills, and deterioration in daily living activities. Each answer is scored from 0-3 according to symptom frequency; “0” for never happened, “1” happened for less than once a week, “2” for once or twice a week, “3” for three times a week or more often. Global score ranges from 0-21; higher values show poor sleep quality. Global score ≥5 demonstrates clinically and significantly poor sleep quality. Diagnostic sensitivity is 89.6%; its specificity is 86.5%.^12^,^13^ Adaptation of PSQI to Turkish patients was done by Agargun et al.^14^

### Assessment of Dietary Intake

Food intake was assessed with dietary records for three consecutive days, being two on weekdays and one on weekend.^15^ Patients were asked to record their food and beverages consumption under supervision from the family; also foods consumed at HD centre or during treatment were observed and recorded by dietitian. Energy and nutrients content was determined with software (E-bispro for Windows, Germany; Turkish version/BeBiS 7).

### Statistical Analysis

Data were analysed with SPSS 22.0. Findings were summarized with descriptive statistics (mean±standard deviation, minimum-maximum, median); chi-square test was used to compare qualitative data. Independent Samples t or Mann Whitney U tests were used for the mean comparison of variables after normality analysis with Shapiro-Wilk test. Logistic Regression Analysis was used to determine variables affecting sleep quality. In producing the binary logistic regression equation, enter method was used to determine the statistical significance of the variables. Wald test statistics and odds ratio (OR) of the regression coefficients were given in tables. Sensitivity, specificity, overall accuracy of the logistic regression models were calculated. p<0.05 was set as statistically significant.

### Ethical Issues

Ethical approval was obtained from Ethical Committee of Eskişehir Yunus Emre State Hospital (number:56761182-903.02.01.524/date:12.05.2015). Research permission was taken from general secretary of Eskişehir Directorate of Public Hospitals (number:84987131-779/179/date:04.06.2015). Patients gave written informed consent.

## RESULTS

Mean age of the participants was 59.19±14.57 years and 51.5% were women. Most of the overall patients composed of housewives (50.5%), retired (23.3%) and tradesmen (11.7%). Patients did not smoke (63.1%) and drink alcoholic beverages (90.3%). Patients had T2DM (27.2%), hypertension (28.2%), hyperlipidaemia (53.4%). Patients were on a 4-hour HD treatment within three times a week. Mean fluid amount between two HD sessions was 2522.3±901.6ml.

Patients (46.6%) defined various sleeping problems; frequent awakening (56.3%), difficulty in falling asleep (43.7%), waking up with respiratory distress (2.1%) and daytime sleepiness (2.1%). Patients did not take any treatment for sleeping problems. Global PSQI score was found to be 7.33±3.94 while 37.9% had good sleep quality (PSQI <5) and 62.1% had poor sleep quality (PSQI ≥5).

Socio-demographic characteristics of patients with good and poor sleep quality were similar. Age and gender did not significantly influence sleep quality (p=0.370, p=0.304; respectively) (Table-I). Appetite had significant impact on sleep quality (p=0.020) (Table-II). Patients with moderate appetite had 3.226 fold higher risk for poor sleep quality compared to patients with good appetite (95% CI; 1.139-9.138), (p=0.027). Also risk for poor sleep quality was 4.194 fold higher in patients with bad appetite compared to patients with good appetite (95% CI; 1.085-16.209), (p=0.038), (Table-II). Specificity and accuracy of the model for appetite were 100% and 62.1%, respectively.

**Table-I:** Socio-demographic and descriptive characteristics of the patients.

*Characteristics*	*Good Sleep Quality (n=39)*		*Poor Sleep Quality (n=64)*		*Total(n=103)*		*p*

	*N*	*%*	*n*	*%*	*n*	*%*
***Gender***						
Male	22	56.4	28	43.8	50	48.5	0.230*
Female	17	43.6	36	56.2	53	51.5
Age (years) (x̄ ± SD)	57.1±15.5 (%95 CI: 52.05-62.15)		60.5±13.8 (%95 CI: 57-63.94)		59.19±14.57 (%95 CI: 56.34-62.03)		0.258**
***Marital Status***						
Married	18	46.2	41	64.1	59	57.3
Single	6	15.4	6	9.4	12	11.7	0.305*
Widowed/Divorced	15	38.4	17	26.6	32	31.0
***Occupation***						
Housewife	17	43.6	35	53.7	52	50.5
Officer	1	2.6	1	1.6	2	1.9
Shepherd	1	2.6	0	0.0	1	1.0	0.642***
Retired	9	23.1	15	23.4	24	23.3
Driver	1	2.6	0	0.0	1	1.0
Unemployed	3	7.7	5	7.8	8	7.8
Musician	2	5.1	1	1.6	3	2.9
Tradesman	5	12.8	7	10.9	12	11.7
***Education Status***						
Illiterate	9	23.1	10	15.6	19	18.4
Literate	3	7.7	10	15.6	13	12.6	0.474***
Primary school	17	43.6	35	54.7	52	50.5
Secondary school	5	12.8	4	6.2	9	8.7
High school	3	7.7	3	4.7	6	5.8
University	2	5.1	2	3.1	4	3.9
***Social Assurance***						
Has	37	94.9	59	92.2	96	93.2	0.707***
Has not	2	5.1	5	7.8	7	6.8
Smoking Status						
Smoking	8	20.5	8	12.5	16	15.5	0.325*
Not smoking	21	53.8	44	68.8	65	63.1
Gave up smoking	10	25.6	12	18.8	22	21.4
***Alcoholic Beverages***						
Drinking	-	-	-	-	-	-
Not drinking	33	84.6	60	93.8	90	90.3	0.173***
Gave up drinking	6	15.4	4	6.2	10	9.7
***Appetite during last week***						
Very bad	-	-	-	-	-	-
Bad	3	7.7	13	20.3	16	15.5	0.017***
Moderate	6	15.4	20	31.2	26	25.2
Good	30	76.9	31	48.4	61	59.2
Very good	-	-	-	-	-	-

* Chi-square test,

** Independent samples t test,

*** Exact method of chi-square test.

**Table-II:** Binary logistic regression analysis of the variables with sleep quality.

*Variable*	*Coefficient*	*Wald*	*p*	*OR*	*95% C.I. for OR*	

					*Lower*	*Upper*
Appetite		7.788	.020			
Appetite (moderate)	1.171	4.860	.027	3.226	1.139	9.138
Appetite (bad)	1.434	4.319	.038	4.194	1.085	16.209
Constant	.033	.016	.898	1.033		
Potassium	-.424	2.150	.143	.654	.371	1.154
Constant	2.560	3.207	.073	12.930		
Creatinine	-.205	3.964	.046	.815	.666	.997
Constant	2.022	6.298	.012	7.555		
Vitamin B1	-2.388	4.542	.033	.092	.010	.826
Constant	1.737	7.679	.006	5.681		
Vitamin B6	-1.528	4.211	.040	.217	.050	.934
Constant	1.680	7.339	.007	5.368		
Folate	-.006	5.030	.025	.994	.988	.999
Constant	1.848	8.227	.004	6.348		

Biochemical findings of poor and good sleeping patients were similar, except for potassium and serum creatinine. Levels of potassium (4.9±0.7 mmol/L) and serum creatinine (8.5±1.6 mg/dl) of good sleepers were significantly higher than of poor sleepers (4.7±0.7 mmol/L, 6.9±2.1 mg/dl; respectively), (p=0.024 and p=0.043, respectively for potassium and serum creatinine differences), (Table-III). As level of serum creatinine increased, risk of having poor sleep quality decreased at 0.815 ratio (95% CI; 0.666-0.997), (p=0.046). Potassium level did not influence sleep quality according to the logistic regression analysis (p=0.143), (Table-II). Specificity, sensitivity and accuracy of the model for potassium were 96.9%, 5.1% and 62.1%. These were determined as 92.2%, 17.9% and 64.1%, respectively for creatinine.

**Table-III:** Biochemical findings of the patients.

*Biochemical Findings*	*Good Sleep Quality (n=39) x̄±SD (min.-max.)*	*Poor Sleep Quality (n=64) x̄±SD (min.-max.)*	*P*
Fasting blood glucose (mg/dl)*	119±43.9 (74-246)		
Median=108	126±76.9 (70-626)		
Median=106.5	0.873		
Sodium (mmol/L)**	136.6±2.7 (132-142)	137.2±3.1 (130-144)	0.517
Potassium (mmol/L)*	4.9±0.7 (3.0-6.3)		
Median=5.00	4.7±0.7 (3.3-7.8)		
Median=4.8	0.024		
Calcium (mg/dL)**	9.1±0.7 (7.7-10.6)	8.6±0.8 (6.7-9.9)	0.082
Phosphorus (mg/dL)*	5.7±1.0 (3.5-7.3)		
Median=5.3	5.2±1.9 (3.2-12.4)		
Median=4.8	0.586		
BUN (mg/dL)**	73.1±14.8 (45-104)	57.7±14.2 (27-78)	0.738
Serum creatinine (mg/dl)**	8.5±1.6 (5.8-11.1)	6.9±2.1 (1.7-10.2)	0.043
Uric acid (mg/dL)**	6.0±0.8 (4.7-8.3)	5.5±0.9 (3.0-7.2)	0.348
MCV*	91.7±16.1 (33.6-102.0)		
Median=93.2	93.9±14.0 (32.0-111.0)		
Median=92.7	0.413		
Iron (mg/dL)**	68.6±30.5 (29-147)	76.0±26.9 (31-141)	0.682
RBC (10^A^6/uL) *	3.6±0.6 (1.2-4.8)		
Median=3.8	3.6±0.5 (2.2-4.9)		
Median=3.6	0.326		
Haemoglobin (g/dL)*	11.3±1.3 (7-13)		
Median=11.7	11.0±1.8 (3-16)		
Median=11.2	0.245		
Haematocrit (%)*	36.5±2.4 (30.1-39.5)		
Median=34.9	34.6±6.9 (10.7-47.1)		
Median=34.0	0.695		
Total Cholesterol (mg/dL)*	173.1±41.5 (112-292)		
Median=158	178.2±42.3 (88-315)		
Median=174	0.314		
HDL-C (mg/dL)**	42.0±13.0 (26-71)	35.1±10.1 (10-59)	0.095
LDL-C (mg/dL)*	96.9±35.1 (49-204)		
Median=88.0	103.4±33.7 (42-214)		
Median=101.5	0.122		
Protein (g/dL)**	6.6±0.5 (5.4-7.8)	6.5±0.6 (5.2-8.3)	0.478
Albumin (g/dL)*	3.6±0.3 (3.0-4.8)		
Median=3.7	3.6±0.4 (2.7-6.0)		
Median=3.6	0.206		

* Mann Whitney U test,

** Independent Samples t test

No difference in intakes of energy, protein, fat, carbohydrate, dietary fibre, vitamins A, E, C and minerals was observed between patients with good and poor sleep quality. Dietary intake of vitamins B_1_ and B_6_ and folate of patients were significantly different. Good sleeping patients consumed significantly higher amounts of above B vitamins (p=0.030, p=0.036, p=0.034; respectively), (Table-IV). Risk of poor sleep quality decreased at ratios of 0.092 (95% CI; 0.010-0.826), (p=0.033), 0.217 (95% CI; 0.050-0.934), (p=0.040) and 0.994 (95% CI; 0.988-0.999), (p=0.025) as dietary vitamins B_1_, B_6_ and folate intake increased, respectively (Table-II). Specificity, sensitivity and accuracy of the model for vitamins B_1_ and B_6_ and folate were 92.2% - 28.2% - 68%, 89.1% - 17.9% - 62.1% and 93.8% - 23.1% - 67%, respectively.

**Table-IV:** Energy and nutrients intake of the patients.

*Energy and Nutrients*	*Good Sleep Quality (n=39) x̄ ± SD (min.-max.)*	*Poor Sleep Quality (n=64) x̄ ± SD (min.-max.)*	*p*
Energy (kcal)	1449±564 (522-2963)	1258±418 (582-3017)	0.104*
	Median=1410	Median=1232	
Protein (g)	52.9±22.4 (20.7-121.0)	47.1±44.5 (18.8-93.4)	0.237*
	Median=49.5	Median=44.5	
Fat (g)	59.9±34.7 (18.1-222.8)	51.4±17.0 (22.7-101.9)	0.251*
	Median=55.0	Median=49.3	
Carbohydrate (g)	171.4±63.5 (67.1-294.6)	149.3±62.8 (38.3-426.1)	0.057*
	Median=160.3	Median=142.6	
Fiber (g)	14.1±6.6 (3.8-34.4)	12.7±5.5 (5.4-30.9)	0.314*
	Median=13.3	Median=11.4	
PUFA (g)	14.4±8.2 (2.9-40.3)	12.6±5.8 (2.8-28.2)	0.455*
	Median=12.1	Median=11.1	
Cholesterol (mg)	222.8±123.6 (18.2-600.2)	210.3±106.0 (17.6-530.9)	0.817*
	Median=214.0	Median=195.2	
Vitamin A (mcg)	953.3±1123.0 (110.8-4833.0)	645.6±538.6 (162.8-3122.4)	0.168*
	Median=550.2	Median=550.2	
Carotene (mg)	1.89±1.99 (0.12-9.9)	1.42±1.36 (0.24-5.96)	0.137*
	Median=1.20	Median=0.97	
Vitamin E (mg)	13.4±7.9 (2.6-38.0)	12.1±5.5 (2.5-27.6)	0.649*
	Median=12.4	Median=11.1	
Vitamin B1 (mg)	0.56±0.21 (0.19-1.02)	0.47±0.16 (0.19-0.90)	0.030**
	Median=0.53	Median=0.47	
Vitamin B2 (mg)	0.99±0.38 (0.31-1.99)	0.85±0.29 (0.44-1.72)	0.084*
	Median=0.95	Median=0.78	
Vitamin B6 (mg)	0.83±0.31 (0.28-1.63)	0.71±0.25 (0.29-1.31)	0.036**
	Median=0.79	Median=0.71	
Folate (mcg)	233.9±91.6 (67.9-441.3)	198.1±62.8 (85.9-407.5)	0.034*
	Median=222.8	Median=188.0	
Vitamin C (mg)	55.2±34.4 (8.3-144.4)	49.3±34.6 (5.5-194.8)	0.329*
	Median=47.4	Median=43.6	
Sodium (mg)	2954.8±1186.4 (921.4-6002.3)	2826.2±985.9 (1120.2-6057.4)	0.418*
	Median=3236.9	Median=2671.7	
Potassium (mg)	1403.6±621.2 (440.7-3285.2)	1195.6±466.0 (489.6-2538.9)	0.098*
	Median=1282.9	Median=1179.4	
Calcium (mg)	455.4±203.8 (87.3-940.0)	432.8±175.2 (164.7-991.5)	0.719*
	Median=397.6	Median=392.0	
Magnesium (mg)	141.4±57.8 (37.1-252.4)	129.7±54.9 (54.3-314.6)	0.231*
	Median=128.6	Median=121.3	
Phosphorus (mg)	762.1±293.2 (242.6-1286.3)	720.0±253.0 (307.2-1398.7)	0.422*
	Median=675.9	Median=673.9	
Iron (mg)	8.2±3.5 (2.2-16.5)	6.8±2.4 (3.2-13.4)	0.060*
	Median=7.7	Median=6.2	
Zinc (mg)	7.2±3.3 (3.1-18.0)	6.5±2.3 (2.9-12.6)	0.444*
	Median=6.4	Median=6.1	

* Mann Whitney U test,

** Independent Samples t test

## DISCUSSION

Sleeping problems are more common among CKD patients than healthy subjects.^2^,^16^ In this study, incidence of poor sleep quality (PSQI>5) was 62.1%. A large number of HD patients (79.0-83.3%) were reported to have one or more sleeping complaints.^17^ Prevalence of poor sleep quality was 83.3% in Kobra et al’s study,^18^ 81.5% in Feride et al’s study,^17^ 73.8% in Sabet et al’s study^19^ which were higher than found in the present study. Factors affecting sleep quality such as depression, sedatives, URL-Synd were excluded in this study, thus prevalence was lower.

Female gender has been found to increase 3.7 times the risk of sleep disorders.^20^ It was argued that difference in anxiety and depression between two genders may be the reason. So, exclusion of patients with depression in this study might have eliminated sleep quality difference between male and female patients. Although a decrease in quality of sleep was shown as people age,^19^ a relation between age and sleep quality was not found in this study. It was reported that younger HD patients had poorer sleep quality.^21^ De Santo et al.^22^ reported that age and sleep disorder were not connected each other. Age may not be decisive factor in sleep quality of HD patients.

Protein catabolism raises and muscle mass and total body protein decreases in CKD patients. Eventually developing uremic scene may cause malnutrition. Malnutrition prevalence changes depending on evaluation criteria in HD patients (18-75%).^23^ According to National Kidney Foundation, serum albumin level <3.4 g/dL and creatinine level <8 mg/dl in HD patients before dialysis treatment; >10% of weight loss or >20% excess of weight; protein intake <0.8 g/kg and energy intake <25 kcal/kg show malnutrition.^24^ Mean serum albumin level of participants was slightly over 3.4 g/dl in both groups (3.6±0.3 g/dl and 3.6±0.4 g/dl, p=0.206) (Table-III). Similarly many studies did not demonstrate a difference.^10^,^21^,^25^,^26^ Serum creatinine level, which is the renal failure criteria and reflects muscle mass and nutritional status, was significantly different in two groups (good vs poor sleepers) (8.5±1.6 mg/dl and 6.9±2.1 mg/dl, respectively; p=0.043), (Table-III). Also risk of poor sleep quality was lower with the increase in serum creatinine levels (p=0.046). In Burrowes et al’s^8^ HEMO cohort, the only significant result showing effect of biochemical indicator on sleep quality was serum creatinine, similarly with this study. In another cohort study, it was again reported that HD patients with higher levels of serum creatinine had better sleep quality.^10^

Nutrition is crucial for HD patients. Adherence to dietary restrictions and avoiding protein-energy deficit should be provided. There are limited papers on sleep quality and food consumption in HD patients who frequently face with sleeping problems.^9^,^10^ In a prospective cohort study (7 years-15 centres-1803 HD patients), patients with poor sleep quality were found to have poor appetite.^10^ In this study, appetite was the major factor affecting sleep quality. Bad appetite caused higher risk of poor sleep quality. The main mechanisms under loss of appetite in CKD are malnutrition and chronic inflammation.^27^ In a study with HD patients, 83.3% of those with protein energy deficit were found to have poor appetite.^28^ Adequate energy and nutrients intake and maintaining nutritional status of these patients may improve quality of sleep by their positive impact on appetite. In this study, good sleeping patients were found to eat adequately, especially having significantly higher intakes of vitamins B_1,_ B_6_ and folate (Table-IV). Inadequate intake of vitamin B_1_ for 21-28 days is known to cause emotional sensitivity, loss of appetite, constipation, nausea/vomiting as well as folate deficiency inducing appetite loss.^29^ There is abundant research about prevalence of B vitamins deficiency in HD patients whereas –to the authors’ knowledge– there is not enough study investigating the association between B vitamins deficiency and sleep. Dietary inadequacy of B vitamins and losing these nutrients by dialysis may cause deficiency-induced appetite loss which may affect sleep quality. Further studies are needed for new nutrition recommendations for HD patients who are already dietary restricted.

Patients with CKD are accepted as poor sleepers; nevertheless a favourable effect of appetite and intake of certain nutrients on sleep quality was proven in this study. Given the consequence that socio-demographic characteristics were not different between poor or good sleepers, in addition to the elimination of physiologic factors influencing sleep quality in CKD and HD patients at baseline; appetite influencing the nutritional status and intake of patients was the major component. In conclusion, this meant that improving nutritional intake of the patient will be able to increase his/her sleep quality. The finding that poor sleeping patients had lower nutrients intake is crucial for dietitians to be aware and patients to well nourish in terms of preventing such a vicious cycle.

### Authors’ Contribution

**DO** conceived, designed and did statistical analysis & editing of manuscript.

**AY** did data collection and manuscript writing.

## References

[1] dos Reis Santos I, Danaga AR, de Carvalho Aguiar I, Oliveira EF, Dias IS, Urbano JJ (2013). Cardiovascular risk and mortality in end-stage renal disease patients undergoing dialysis:Sleep study, pulmonary function, respiratory mechanics, upper airway collapsibility, autonomic nervous activity, depression, anxiety, stress and quality of life:A prospective, double blind, randomized controlled clinical trial. BMC Nephrol.

[2] Maniam R, Subramanian P, Singh SKS, Lim SK, Chinna K, Rosli R (2014). Preliminary study of an exercise programme for reducing fatigue and improving sleep among long-term haemodialysis patients. Singapore Med J.

[3] Uzun Ş, Kara B, Işcan B (2003). Sleep disorders in hemodialysis patients with chronic renal failure. J Turk Soc Nephrol.

[4] Russcher M, Koch BCP, Nagtegaal JE, van Ittersum FJ, Pasker-de Jong PCM, Hagen EC (2013). Long-term effects of melatonin on quality of life and sleep in haemodialysis patients (Melody study):A randomized controlled trial. Br J Clin Pharmacol.

[5] Gade K, Blaschke S, Rodenbeck A, Becker A, Anderson-Schmidt H, Cohrs S (2013). Uremic restless legs syndrome and sleep quality in patients with end-stage renal disease on hemodialysis:Potential role of homocysteine and parathyroid hormone. Kidney Blood Press Res.

[6] Koenig P, Nagl C, Neurauter G, Schennac H, Brandacher G, Fuchs D (2010). Enhanced degradation of tryptophan in patients on hemodialysis. Clin Nephrol.

[7] Zhang J, Wang C, Gong W, Peng H, Tang Y, Li CC (2014). Association between sleep quality and cardiovascular damage in pre-dialysis patients with chronic kidney disease. BMC Nephrol.

[8] Burrowes JD, Russell GB, Unruh M, Rocco MV (2012). Is nutritional status associated with self-reported sleep quality in the HEMO Study Cohort?. J Renal Nutr.

[9] Köse E, Turgutalp K, Kıykım A, Celik F (2014). The association between feeding habits, nutritional parameters and quality of sleep in hemodialysis patients. Turk Neph Dial Transpl.

[10] Burrowes J, Russell G, Unruh M, Rocco M (2012). Nutrition-Related predictors of sleep duration in hemodialysis patients. Kidney Res Clin Pract.

[11] Zabel R, Ash S, King N, Juffs P, Bauer J (2012). Relationships between appetite and quality of life in hemodialysis patients. Appetite.

[12] Buysse DJ, Reynolds CF, Monk TH, Berman SR, Kupfer DJ (1989). The Pittsburgh sleep quality index:A new instrument for psychiatric practice and research. Psychiatry Res.

[13] Buysse DJ, Reynolds CF, Monk TH, Hoch CC, Yeager AL, Kupfer DJ (1991). Quantification of subjective sleep quality in healthy elderly men and women using the Pittsburgh Sleep Quality Index (PSQI). Sleep.

[14] Agargun MY, Kara H, Anlar O (1996). Pittsburgh Uyku Kalitesi Indeksinin geçerliği ve guvenirliği. Turk Psikiyatri Derg.

[15] Lee RD, Nieman DC (2003). Nutritional Assessment Third Edition.

[16] Cölbay M, Yuksel Ş, Fidan F, Acarturk G, Karaman Ö, Ünlu M (2007). Evaluation of the hemodialysis patient with Pittsburgh sleep quality index. Tuberkuloz ve Toraks Dergisi.

[17] Sert F, Demir AB, Bora I, Yıldız A, Ocakoğlu G, Ersoy A (2015). The Research of Sleep Disorders and Their Effects on Quality of Life in Patients with Chronic Renal Failure and Renal Transplant. J Turk Sleep Med.

[18] Parvan K, Lakdizaji S, Roshangar F, Mostofi M (2013). Quality of sleep and its relationship to quality of life in hemodialysis patients. J Caring Sci.

[19] Sabet R, Naghizadeh MM, Azari S (2012). Quality of sleep in dialysis patients. Iran J Nurs Midwifery Res.

[20] Celik G, Annagur BB, Yılmaz M, Kara F (2012). Findings of multidimensional instruments for determining psychopathology in diabetic and non-diabetic hemodialysis patients. Int J Clin Exp Med.

[21] Unruh M, Tamura MK, Larive B, Rastogi A, James S, Schiller B (2011). Impact of sleep quality on cardiovascular outcomes in hemodialysis patients:Results from the frequent hemodialysis network study. Am J Nephrol.

[22] De Santo RM, Bartiromo M, Cesare CM, Cirillo M (2008). Sleep disorders occur very early in chronic kidney disease. J Nephrol.

[23] Ünal HÜ, Korkmaz M, Selçuk H (2010). Malnutrition Pathogenesis and Evaluation in Chronic Kidney Disease patients. Guncel Gastroenteroloji.

[24] Kopple JD (2000). Rationale for an International Federation of Kidney Foundations. Am J Kidney Dis.

[25] Guney I, Atalay H, Solak Y, Altıntepe L, Toy H, Tonbul HZ (2010). Predictors of sleep quality in hemodialysis patients. Int J Artif Organs.

[26] Pai MF, Hsu SP, Yang SY, Ho TI, Lai CF, Peng YS (2007). Sleep disturbance in chronic hemodialysis patients:the impact of depression and anemia. Ren Fail.

[27] Doğukan A, Ulaç C (1998). Chronic kidney failure and leptin. Journal of The Turkish Nephrology.

[28] Sahathevan S, Se CH, Hoe Ng S, Chinna K, Harvinder GS, Chee WSS (2015). Assessing protein energy wasting in a Malaysian haemodialysis population using self-reported appetite rating:A cross-sectional study. BMC Nephrol.

[29] Corken M, Poter J (2011). Is vitamin B_6_ deficiency an under-recognized risk in patients receiving haemodialysis?A systematic review 2000-2010. Nephrology.

